# Maternal and Fetal Outcomes in Early-Onset vs. Late-Onset Preeclampsia: A Retrospective Study at a Tertiary Care Institute in India

**DOI:** 10.7759/cureus.102711

**Published:** 2026-01-31

**Authors:** Uditi S Harjai, Ketaki K Junnare

**Affiliations:** 1 Obstetrics and Gynaecology, Smt. Kashibai Navale Medical College and General Hospital, Pune, IND

**Keywords:** fetal development, maternal health, neonatal outcomes, preeclampsia, pregnancy complications

## Abstract

Background

Preeclampsia is a hypertensive disorder of pregnancy associated with significant maternal and perinatal morbidity and mortality. Distinguishing between early- and late-onset preeclampsia is crucial due to differences in pathophysiology, severity, and outcomes. This study aimed to evaluate and contrast clinical characteristics, complications, and outcomes between the two types.

Aim & objective

The aim of the study is to compare the maternal and fetal outcomes in pregnancies complicated by early-onset preeclampsia (before 34 weeks) versus late-onset preeclampsia (34 weeks or later) and identify specific clinical risks and prognostic implications associated with the timing of disease onset.

Materials & methods

A hospital-based retrospective observational study was conducted at Smt. Kashibai Navale Medical College and General Hospital, Pune, from November 2022 to October 2024. A total of 88 pregnant women diagnosed with preeclampsia were categorized into early-onset (<34 weeks) and late-onset (≥34 weeks) groups. Clinical data were extracted from medical records and analyzed using chi-square and Fisher’s exact tests to assess associations between disease onset and maternal/neonatal outcomes.

Results

Among the 88 cases, early-onset and late-onset preeclampsia were nearly evenly distributed (48.9% vs. 51.1%). Severe preeclampsia was significantly more frequent in the early-onset group (93.0% vs. 62.2%, p=0.001). Early-onset preeclampsia was strongly associated with the cases who underwent IVF (in vitro fertilization) treatment (p=0.001). Early-onset cases had significantly higher rates of preterm delivery (<34 weeks: 76.7%), extremely low birth weight (<1.5 kg: 65.1%), intrauterine death (20.9%, p=0.002), NICU (Neonatal Intensive Care Unit) admissions (p<0.001), and low APGAR scores (p<0.001). Maternal complications were more frequent in early-onset cases, although the difference was not statistically significant.

Discussion

The study affirms that early-onset preeclampsia is associated with great severity disease and significantly poorer neonatal outcomes, especially in terms of prematurity, fetal growth restriction, and neonatal morbidity. IVF emerged as a notable risk factor for early-onset pre-eclampsia. Despite higher complication rates, maternal mortality was zero in this cohort.

Conclusion

Early-onset preeclampsia poses greater risks to both mother and fetus than late-onset disease. Identifying high-risk pregnancies, especially IVF conceptions, and implementing timely interventions are essential to improving outcomes. Enhanced surveillance and preparedness of neonatal intensive care are particularly critical in early-onset cases.

## Introduction

Preeclampsia is a complex hypertensive disorder of pregnancy that affects approximately 2-8% of all pregnancies worldwide and remains one of the leading causes of maternal and perinatal morbidity and mortality globally [1]. This multifaceted condition is characterized by new-onset hypertension (blood pressure ≥140/90 mmHg) and proteinuria (≥300 mg/24 hours) after 20 weeks of gestation, often accompanied by multiorgan dysfunction affecting the liver, kidneys, brain, and cardiovascular system [2]. The disorder demonstrates significant heterogeneity in its clinical presentation, timing of onset, and severity, which has important implications for both maternal and fetal outcomes.

The classification of preeclampsia into early-onset (occurring before 34 weeks of gestation) and late-onset (occurring at or after 34 weeks) forms is clinically meaningful and reflects distinct pathophysiological mechanisms [3]. Early-onset preeclampsia is typically associated with more severe maternal complications such as eclampsia, hemolysis, elevated liver enzymes, and low platelet count (HELLP) syndrome, acute renal failure, pulmonary edema, and cerebral hemorrhage [4]. These severe maternal manifestations are thought to result from underlying placental insufficiency due to abnormal trophoblast invasion and spiral artery remodeling in early pregnancy, leading to widespread endothelial dysfunction [4].

Conversely, late-onset preeclampsia may have different etiological pathways, with stronger associations with maternal pre-pregnancy obesity, chronic hypertension, and metabolic syndrome. While late-onset cases generally have better maternal outcomes compared to early-onset cases, they still carry significant risks for both mother and fetus, particularly due to the higher likelihood of preterm delivery and associated neonatal complications [5].

The fetal consequences of preeclampsia vary substantially between early and late onset forms. Early-onset preeclampsia is frequently complicated by intrauterine growth restriction (IUGR), placental abruption, and extremely preterm birth, leading to higher rates of perinatal mortality and morbidity including respiratory distress syndrome, intraventricular hemorrhage, and necrotizing enterocolitis [5]. Late-onset preeclampsia, while generally associated with less severe placental dysfunction, still results in increased rates of preterm delivery, cesarean section, and neonatal intensive care unit admission compared to normotensive pregnancies [6].

The rationale for this comprehensive study stems from the critical need to better understand the distinct clinical trajectories and outcomes of early-onset versus late-onset preeclampsia. By systematically comparing maternal and fetal outcomes between these two groups, we aim to identify specific risk factors, develop more targeted interventions, and improve prognostic accuracy. This distinction is crucial for clinical decision-making, particularly regarding timing of delivery, intensity of maternal monitoring, and preparation for neonatal intensive care. Furthermore, elucidating the differences between these forms of preeclampsia may provide insights into their distinct pathophysiological mechanisms, potentially leading to the development of novel biomarkers and therapeutic approaches.

## Materials and methods

This retrospective observational study was carried out at Smt. Kashibai Navale Medical College and General Hospital (SKNMC & GH), a tertiary care teaching hospital located in Narhe, Pune. The study period spanned from November 2022 to October 2024 and included all pregnant women diagnosed with preeclampsia who presented to the antenatal outpatient department (OPD), labor ward, or were admitted to the hospital during this time.

Inclusion and exclusion criteria

Inclusion Criteria

All pregnant women diagnosed with preeclampsia during the study period were included, irrespective of parity or gestational age at presentation. Preeclampsia was diagnosed and classified according to the International Society for the Study of Hypertension in Pregnancy (ISSHP) guidelines [7]. Only patients with complete and accessible medical records, including antenatal, intrapartum, and neonatal details, were considered eligible for analysis.

Exclusion Criteria

Women with pre-existing chronic hypertension diagnosed prior to pregnancy or before 20 weeks of gestation, diabetes mellitus (pre-gestational or gestational), chronic kidney disease or other renal disorders predating pregnancy, autoimmune or connective tissue disorders, pre-existing cardiovascular disease, multiple gestations, and known fetal congenital anomalies were excluded from the study. Patients with incomplete or missing medical records were also excluded to ensure data accuracy and reliability.

Classification of study groups

Patients were categorized into early-onset and late-onset preeclampsia groups based on the gestational age at the time of diagnosis, in accordance with ISSHP guidelines [7]. Early-onset preeclampsia is defined as diagnosis before 34 weeks of gestation, while late-onset preeclampsia is defined as diagnosis at or after 34 weeks. Women diagnosed with early-onset preeclampsia who were managed expectantly and subsequently delivered after 34 weeks were analyzed under the early-onset preeclampsia group, as classification was based on the timing of disease onset rather than gestational age at delivery.

Eligible patients were categorized into two groups based on the gestational age at which preeclampsia was diagnosed. According to the International Society for the Study of Hypertension in Pregnancy (ISSHP) guidelines [7], Group A was made up of women who had early-onset preeclampsia, which is defined as the onset of the condition before 34 weeks of gestation. Group B was made up of women who had late-onset preeclampsia, which is defined as the onset of the condition at or after 34 weeks of gestation. All cases that met the inclusion criteria during the study period were included, making the sample size open-ended. 

Data were retrospectively retrieved from institutional medical records, including hospital registers, antenatal care (ANC) and postnatal care (PNC) ward files, labor room records, and neonatal intensive care unit (NICU) documentation. All extracted data were fully de-identified prior to analysis, with patient names, hospital identification numbers, and other personal identifiers removed and replaced with coded study numbers to ensure confidentiality.

The information collected included when preeclampsia started (either early before 34 weeks or late at 34 weeks or later), how severe it was (either severe or not severe), and complications before delivery (like eclampsia, placental abruption). Severity of preeclampsia was classified according to ISSHP criteria. Severe preeclampsia is defined by the presence of one or more of the following: systolic blood pressure ≥160 mmHg and/or diastolic blood pressure ≥110 mmHg on at least two occasions; evidence of end-organ dysfunction such as renal impairment (serum creatinine ≥1.1 mg/dL or doubling of baseline), hepatic involvement (elevated liver transaminases ≥2 times normal, severe persistent right upper quadrant or epigastric pain), neurological complications (eclampsia, altered sensorium, visual disturbances), hematological abnormalities (platelet count <100,000/µL), pulmonary edema, or uteroplacental dysfunction. Non-severe preeclampsia included cases that did not meet the above criteria.

Pregnancy outcomes comprised gestational age at delivery, categorized as early preterm (<34 weeks), late preterm (34-36 weeks), and term (≥37 weeks), as well as mode of delivery, including vaginal birth and lower segment cesarean section (LSCS).

Postpartum complications were also documented, such as the need for blood transfusion (BT), postpartum hemorrhage (PPH), disseminated intravascular coagulation (DIC), and postpartum eclampsia. The neonatal parameters assessed encompassed birth weight, classified into four categories (>2.5 kg, 2.0-2.5 kg, 1.5-2.0 kg, and <1.5 kg), APGAR scores at one and five minutes, necessity for NICU admission, and perinatal mortality outcomes including intrauterine death and fresh stillbirth (FSB).

Statistical analysis was performed using appropriate tests. Descriptive statistics summarized clinical and demographic characteristics. A comparison between the early- and late-onset preeclampsia groups was done using Pearson’s chi-square test or Fisher’s exact test, depending on what was suitable, to look at the relationships between when the disease started and factors like severity, maternal complications, gestational age at delivery, neonatal outcomes,

Ethical clearance for this study was obtained from the Institutional Review Board of SKNMC & GH. The study adhered strictly to the principles of the Declaration of Helsinki. As this was a retrospective study using de-identified medical records, the requirement for individual informed consent was waived by the ethics committee.

## Results

A total of 88 women diagnosed with preeclampsia were included in the study. The mean age distribution showed that the majority of women were ≤30 years (73.9%), while 26.1% were above 30 years of age. Primigravidae constituted 55.7% of the cohort. Most pregnancies were spontaneously conceived (89.8%), with 10.2% resulting from IVF (in vitro fertilization).

Chronic medical comorbidities were relatively uncommon; diabetes mellitus was present in 4.5% and chronic hypertension in 5.7% of cases. A history of pregnancy-induced hypertension (PIH) was observed in 21.6% of women.

With regard to disease characteristics, early-onset preeclampsia accounted for 48.9% of cases, while late-onset disease constituted 51.1%. Severe preeclampsia was the predominant presentation, observed in 77.3% of cases. These baseline characteristics are summarized in Table 1.

**Table 1:** Baseline demographic and clinical characteristics of the study population (n = 88) PIH: Pregnancy-induced hypertension

Characteristic	Category	Frequency (n)	Percentage (%)
Age (years)	≤30	65	73.9
	>30	23	26.1
Parity	Primigravida	49	55.7
	Multigravida	39	44.3
Mode of Conception	Spontaneous	79	89.8
	IVF	9	10.2
Diabetes Mellitus	Yes	4	4.5
	No	84	95.5
Chronic Hypertension	Yes	5	5.7
	No	83	94.3
History of PIH	Yes	19	21.6
	No	69	78.4
Time of Onset of Preeclampsia	Early-onset	43	48.9
	Late-onset	45	51.1
Severity of Preeclampsia	Severe	68	77.3
	Non-severe	20	22.7

Among the 88 women diagnosed with preeclampsia, the onset was almost evenly split, with 43 (48.9%) experiencing early-onset preeclampsia and 45 (51.1%) presenting with late-onset disease. The majority of cases (68; 77.3%) were classified as severe preeclampsia, while only 20 (22.7%) were non-severe, highlighting a high burden of severe disease in the cohort. The findings indicate that severe preeclampsia was more common than non-severe forms in this population. Although late-onset preeclampsia was marginally more prevalent, early-onset cases constituted nearly half of the total burden (Table 2). 

**Table 2:** Distribution of study participants based on severity and onset of preeclampsia (n = 88)

Characteristic	Frequency (n)	Percentage (%)
Time of Onset		
Early Onset	43	48.9
Late Onset	45	51.1
Severity		
Severe Preeclampsia	68	77.3
Non-severe Preeclampsia	20	22.7

The association between various maternal risk factors and the time of onset of preeclampsia was assessed among 88 women diagnosed during the study period. Most risk factors, including overt diabetes mellitus, advanced maternal age (>30 years), multiparity, chronic hypertension, and history of PIH, did not show any statistically significant association with whether preeclampsia was of early or late onset. The p-values for these variables ranged from 0.264 to 0.963, indicating no meaningful difference. Maternal age did not show a significant association with the timing of onset of preeclampsia. Although a higher proportion of women were ≤30 years of age, the distribution of early- and late-onset preeclampsia was comparable across age groups (p = 0.551), indicating that maternal age was not a significant determinant of disease onset in this cohort. There was a statistically significant association between the time of onset of preeclampsia and neonatal birth weight (p < 0.001). Among early-onset cases (n = 43), a large proportion of neonates (28; 65.1%) had extremely low birth weight (<1.5 kg), and only one neonate (2.3%) weighed more than 2.5 kg. In contrast, none of the late-onset group (n = 45) had babies weighing less than 1.5 kg, and 20 neonates (44.4%) had normal birth weight (>2.5 kg). The difference was highly significant on both the Pearson chi-square and the likelihood ratio tests. Early-onset preeclampsia is significantly associated with lower neonatal birth weights, especially extremely low birth weight (<1.5 kg), compared to late-onset cases. This emphasizes the greater fetal growth restriction and placental insufficiency seen in early-onset preeclampsia, reinforcing the need for close fetal surveillance and timely delivery in such cases. However, a significant association was found with IVF pregnancies. In IVF-conceived pregnancies with preeclampsia, all developed early-onset, and this association was statistically significant (p = 0.001) (Table 3). This finding suggests that IVF may be an important predictor for early-onset preeclampsia, likely due to underlying maternal or placental factors linked to assisted reproduction. In contrast, other conventional risk factors did not demonstrate predictive value for the timing of disease onset in this cohort.

**Table 3:** Association between risk factors and time of onset of preeclampsia (n = 88) PIH: Pregnancy-induced hypertension ** denotes a significant value

Risk Factor	Category	Early Onset (n=43)	Late Onset (n=45)	Total (n=88)	Test Used	Test value	p-value
Overt Diabetes Mellitus	No	41	43	84	Likelihood Ratio	0.002	0.963
	Yes	2	2	4			
Age >30	No	32	33	65	Pearson Chi-Square	0.013	0.551
	Yes	11	12	23			
Multiparity	Multi	20	19	39	Pearson Chi-Square	0.164	0.425
	Primi	23	26	49			
Hypertension	No	41	42	83	Pearson Chi-Square	0.167	0.522
	Yes	2	3	5			
IVF	No	34	45	79	Pearson Chi-Square	13.968	0.001**
	Yes	9	0	9			
History of PIH	No	32	37	69	Pearson Chi-Square	0.791	0.264
	Yes	11	8	19			

Among the 88 preeclampsia cases, early-onset preeclampsia was significantly more likely to be severe than late-onset cases. Out of the 43 early-onset cases, 40 (93.0%) had severe preeclampsia, compared to 28 (62.2%) of the 45 late-onset cases. Conversely, non-severe preeclampsia was more frequent in the late-onset group (17 vs 3 cases). This association was statistically significant (Pearson χ² = 11.878, p = 0.001; Fisher’s exact test p = 0.001), indicating that early-onset preeclampsia is significantly associated with increased severity compared to late-onset disease (Table 4). The maternal complications were compared; while there were many maternal comorbidities, zero maternal mortality was noted in our study.

**Table 4:** Association between time of onset of preeclampsia and severity

Severity of Preeclampsia	Early Onset (n = 43)	Late Onset (n = 45)	Total (n = 88)	χ² Value	p-value
Severe	40	28	68	11.878	0.001
Non-severe	3	17	20		
Total	43	45	88		

In the present study, maternal complications among women diagnosed with preeclampsia were analyzed based on the time of onset, early (n = 43) versus late (n = 45). Eclampsia was observed equally in both groups, affecting five women each. Other complications such as placental abruption, HELLP syndrome, and combined complications (i.e., occurrence of more than one among HELLP, abruption, or eclampsia) were more frequently associated with early-onset preeclampsia. Specifically, three cases of abruption and five cases of HELLP syndrome were reported in the early-onset group, compared to 1 and 1, respectively, in the late-onset group. One patient in the early-onset group also presented with combined complications, while none in the late-onset group did. Despite these numerical differences suggesting that early-onset preeclampsia may be associated with more severe maternal complications, the association did not reach statistical significance (p = 0.236, Fisher’s exact test) (Table 5). Therefore, this study did not statistically confirm the apparent higher risk for adverse maternal outcomes associated with early-onset cases.

**Table 5:** Association between time of onset of preeclampsia and antepartum complications (n = 88)

Time of Onset	Eclampsia	Abruption	HELLP	Combined (HELLP/Abruption/Eclampsia)	No Complication	Total	Test of Significance	p-value
Early Onset	5	3	5	1	29	43	Fisher’s Exact	0.236
Late Onset	5	1	1	0	38	45		
Total	10	4	6	1	67	88		

Maternal hemorrhagic and related complications were comparable between the two groups. Among women with early-onset preeclampsia (n = 43), six required BT, one developed DIC requiring transfusion, and one had eclampsia with BT. A similar distribution of BT requirement was observed in the late-onset preeclampsia group, indicating no significant difference between the groups with respect to hemorrhagic complications. In contrast, in the late-onset group (n = 45), five women required BT and one had postpartum hemorrhage (BT/PPH); no cases of DIC, eclampsia, or combined complications were reported in this group. Although complications such as DIC and eclampsia appeared to occur exclusively in early-onset preeclampsia, this distribution did not reach statistical significance (p = 0.525, Fisher’s exact test) (Table 6). Hence, while the early-onset group showed a wider spectrum and higher frequency of severe hemorrhagic complications, the difference was not statistically significant.

**Table 6:** Association between the time of onset of preeclampsia and postpartum complications (n = 88) BT: Blood transfusion; PPH: postpartum hemorrhage; DIC: disseminated intravascular coagulation

Time of Onset	BT	BT/PPH	DIC/BT	Eclampsia	Eclampsia/BT	No Complication	Total	Test of Significance	p-value
Early Onset	6	0	1	1	1	34	43	Fisher’s Exact Test	0.525
Late Onset	5	1	0	0	0	39	45		
Total	11	1	1	1	1	73	88		

A strong and statistically significant association was observed between the time of onset of preeclampsia and gestational age at delivery (p < 0.001). Among the 43 women with early-onset preeclampsia, the majority (33; 76.7%) delivered in the early preterm period (<34 weeks), and only two (4.7%) reached term. In contrast, of the 45 women with late-onset preeclampsia, 29 (64.4%) delivered at term. The likelihood ratio tests confirmed this significant relationship (Table 7). Early-onset preeclampsia is strongly associated with earlier delivery, particularly before 34 weeks, whereas late-onset cases are more likely to progress to term. This highlights the clinical severity and need for earlier intervention in early-onset preeclampsia.

**Table 7:** Association between time of onset of preeclampsia and gestational age at delivery (n = 88)

Gestational Age at Delivery	Early Onset (n = 43)	Late Onset (n = 45)	Total (n = 88)	Test	p-value
Early Preterm (<34 weeks)	33	0	33	Likelihood Ratio = 76.564	<0.001
Late Preterm (34–36 weeks)	8	16	24		
Term (≥37 weeks)	2	29	31		
Total	43	45	88		

The mode of delivery was compared between women with early-onset and late-onset preeclampsia. Among the 88 women studied, 38 (43.2%) delivered vaginally and 50 (56.8%) underwent LSCS. In the early-onset group, 20 women (46.5%) had vaginal delivery and 23 (53.5%) underwent LSCS. Similarly, in the late-onset group, 18 women (40.0%) delivered vaginally while 27 (60.0%) required LSCS. Statistical analysis using the chi-square test showed no significant association between the time of onset of preeclampsia and mode of delivery (χ² = 0.38, p = 0.537). This indicates that the likelihood of cesarean or vaginal delivery was comparable between early- and late-onset preeclampsia (Table 8). 

**Table 8:** Association between time of onset of preeclampsia and mode of delivery (n = 88) LSCS: Lower segment cesarean section

Mode of Delivery	Early Onset (n=43)	Late Onset (n = 45)	Total (n=88)	Test	p-value
Vaginal Delivery	20	18	38	Chi square = 0.38	0.537
LSCS	23	27	50		
Total	43	45	88		

There was a statistically significant association between the time of onset of preeclampsia and neonatal birth weight (p < 0.001). Among early-onset cases (n = 43), a large proportion of neonates (28; 65.1%) had extremely low birth weight (<1.5 kg), and only one neonate (2.3%) weighed more than 2.5 kg. In contrast, none of the late-onset group (n = 45) had babies weighing less than 1.5 kg, and 20 (44.4%) had normal birth weight (>2.5 kg). The difference was highly significant on likelihood ratio tests. Early-onset preeclampsia is significantly associated with lower neonatal birth weights, especially extremely low birth weight (<1.5 kg), compared to late-onset cases (Table 9). This highlights the greater risk of fetal restricted growth and problems with the placenta in early-onset preeclampsia, emphasizing the need for careful monitoring of the baby and timely delivery in these cases.

**Table 9:** Association between time of onset of preeclampsia and birth weight (n = 88)

Birth Weight (kg)	Early Onset (n = 43)	Late Onset (n = 45)	Total (n = 88)	Test	p-value
>2.5	1	20	21	Likelihood Ratio = 66.624	<0.001
<1.5	28	0	28		
1.5 to 2.0	11	12	23		
2.0 to 2.5	3	13	16		
Total	43	45	88		

Among the 88 preeclamptic women studied, FSB occurred in one case (1.1%) in the late-onset group, with no statistically significant association between time of onset and FSB (p = 0.245). Intrauterine death was significantly more frequent in early-onset preeclampsia (nine cases, 20.9%) compared to late-onset disease (two cases, 4.4%) (p = 0.002). Although a higher number of neonates born to women with late-onset preeclampsia required NICU admission (n = 44) compared to those with early-onset disease (n = 29), this difference reflects institutional admission practices and survival patterns rather than disease severity alone (p < 0.001) (Table 10).

**Table 10:** Association between time of onset of preeclampsia and neonatal outcomes (n = 88)

Neonatal Outcome	Early Onset (n = 43)	Late Onset (n = 45)	Total (n = 88)	Test	p-value
Fresh Stillbirth (FSB) Seen					
No	43	44	87	Likelihood Ratio = 1.352	0.245
Yes	0	1	1		
Intrauterine Death (IUD)					
No	34	43	77	Pearson Chi-Square = 12.013	0.002
Yes	9	2	11		
NICU Admission					
NICU Required	29	44	73	Pearson Chi-Square = 14.311	<0.001
No NICU	14	1	15		

A statistically significant association existed between the time of onset of preeclampsia and APGAR scores at one minute (p < 0.001). Early-onset preeclampsia was linked with lower APGAR scores and a higher incidence of APGAR = 0 at one minute. A significant association was also observed at five minutes (p = 0.006). Infants born to early-onset preeclampsia cases were more likely to have APGAR <7 or 0 at five minutes, indicating poorer neonatal outcomes (Table 11).

**Table 11:** Association between time of onset of preeclampsia and APGAR scores (n = 88) APGAR: Appearance (skin color), Pulse (heart rate), Grimace (reflex irritability), Activity (muscle tone), and Respiration (breathing effort)

APGAR Score	Early Onset (n = 43)	Late Onset (n = 45)	Total (n = 88)	Test	p-value
At 1 Minute					
>7	11	32	43	Pearson Chi-Square = 18.341	<0.001
<7	23	10	33		
0	9	3	12		
At 5 Minutes					
>7	27	41	68	Pearson Chi-Square = 10.342	0.006
<7	7	1	8		
0	9	3	12		
Total	43	45	88		

## Discussion

During the study period, a total of 3,498 deliveries occurred at our institution, of which 88 women were diagnosed with preeclampsia, yielding an overall incidence of 2.5%. This incidence is comparable to the globally reported prevalence of preeclampsia, ranging from 2 to 8% in similar tertiary care settings [8]. The predominance of severe preeclampsia (77.3%) and nearly equal split between early- (48.9%) and late-onset (51.1%) disease underscores the significant clinical burden of hypertensive disorders in this population.

The only significant risk factor associated with early-onset preeclampsia was IVF conception, where all nine of the IVF pregnancies with preeclampsia had early-onset disease (p=0.001). This finding supports prior research indicating a strong association between IVF (particularly fresh embryo transfers) and early, severe preeclampsia due to placental insufficiency and defective placentation [9-11]. Population studies have also reported an elevated risk of hypertensive disorders, prematurity, low birthweight, and cesarean delivery among IVF-conceived pregnancies [12].

Our results reinforce the established observation that early-onset preeclampsia is linked to more adverse maternal and neonatal outcomes. Though antepartum and postpartum maternal complications were more frequent in early-onset cases, these did not reach statistical significance-likely due to sample size constraints. Nevertheless, large cohort studies have consistently demonstrated higher rates of severe features, prolonged hospitalization, and end-organ dysfunction in early-onset preeclampsia [8,13]. Our study shows zero cases of maternal mortality.

Neonatal outcomes exhibited stark contrasts. Stillbirth occurred significantly more in the early-onset group (20.9% vs 4.4%; p=0.002), echoing findings from low-resource settings where early-onset preeclampsia correlated with higher perinatal mortality [8,14]. NICU admissions were also significantly greater in early onset (p<0.001), reflecting the increased need for neonatal support due to prematurity [8,15].

Crucially, APGAR scores at both one and five minutes were markedly lower in the early-onset group (p<0.001 and p=0.006, respectively), consistent with prior reports linking severe preeclampsia to suboptimal neonatal adaptation and higher rates of low APGAR [13,16]. This trend likely stems from earlier gestational age at delivery and IUGR, known to accompany early-onset disease.

Our study further confirmed that early-onset preeclampsia is strongly associated with preterm delivery (<34 weeks in 76.7% vs none in late-onset) and significantly reduced neonatal birthweights (65.1% <1.5 kg vs none in late-onset; both p<0.001). These findings are in agreement with large multicenter analyses demonstrating that early-onset pre-eclampsia is a key driver of preterm birth and low birthweight [8,17]. The significantly lower neonatal birth weight observed in early-onset preeclampsia may not be attributable to prematurity alone but also reflects the underlying placental dysfunction and fetal growth restriction commonly associated with severe forms of the disease. Impaired uteroplacental perfusion and abnormal trophoblastic invasion in early-onset preeclampsia are well-recognized mechanisms contributing to IUGR and low birth weight.

The limitations of this study include its single-center setting and relatively limited sample size, which may have reduced the power to detect differences in certain less frequent maternal and neonatal complications. However, the standardized diagnostic criteria and uniform institutional management protocols strengthen the validity of the findings.

## Conclusions

This study supports existing literature in demonstrating that early-onset preeclampsia is associated with significantly poorer neonatal outcomes, including higher rates of stillbirths, NICU admissions, low APGAR scores, preterm birth, and low birthweight compared with late-onset disease. Furthermore, IVF emerges as a notable risk factor for early-onset preeclampsia. Efforts to enhance antenatal surveillance, early detection (potentially using biomarkers such as sFlt-1/PlGF), and timely interventions are warranted to improve outcomes in high-risk preeclamptic pregnancies.

## References

[1] Karrar SA, Martingano DJ, Hong PL (2025). Preeclampsia. StatPearls [Internet].

[2] Lambert G, Brichant JF, Hartstein G, Bonhomme V, Dewandre PY (2014). Preeclampsia: an update. Acta Anaesthesiol Belg.

[3] Phipps EA, Thadhani R, Benzing T, Karumanchi SA (2019). Pre-eclampsia: pathogenesis, novel diagnostics and therapies. Nat Rev Nephrol.

[4] Khalid F, Mahendraker N, Tonismae T (2025). HELLP Syndrome. StatPearls [Internet].

[5] Chang KJ, Seow KM, Chen KH (2023). Preeclampsia: recent advances in predicting, preventing, and managing the maternal and fetal life-threatening condition. Int J Environ Res Public Health.

[6] Guida JP, Surita FG, Parpinelli MA, Costa ML (2017). Preterm preeclampsia and timing of delivery: a systematic literature review. Rev Bras Ginecol Obstet.

[7] Magee LA, Brown MA, Hall DR (2022). The 2021 International Society for the Study of Hypertension in Pregnancy classification, diagnosis & management recommendations for international practice. Pregnancy Hypertens.

[8] Abalos E, Cuesta C, Grosso AL, Chou D, Say L (2013). Global and regional estimates of preeclampsia and eclampsia: a systematic review. Eur J Obstet Gynecol Reprod Biol.

[9] Teka H, Yemane A, Abraha HE (2023). Clinical presentation, maternal-fetal, and neonatal outcomes of early-onset versus late onset preeclampsia-eclampsia syndrome in a teaching hospital in a low-resource setting: a retrospective cohort study. PLoS One.

[10] Niu Y, Suo L, Zhao D (2023). Is artificial endometrial preparation more associated with early-onset or late-onset preeclampsia after frozen embryo transfer?. J Assist Reprod Genet.

[11] Gui J, Ling Z, Hou X, Fan Y, Xie K, Shen R (2020). In vitro fertilization is associated with the onset and progression of preeclampsia. Placenta.

[12] Dai F, Lan Y, Pan S, Wang Y, Hua Y, Xiao W (2023). Pregnancy outcomes and disease phenotype of hypertensive disorders of pregnancy in singleton pregnancies after in vitro fertilization: a retrospective analysis of 1130 cases. BMC Pregnancy Childbirth.

[13] Rahman L, Anwar R, Mose JC (2024). Maternal and neonatal outcome among women with early-onset preeclampsia and late-onset preeclampsia. Hypertens Pregnancy.

[14] Atluri S, Nandish S, Manol J (2017). Comparative study of maternal and perinatal outcome in early onset and late onset preeclampsia. J Evid Based Med Healthc.

[15] Thakur N, Thakur S, Pathania K, Thakur R (2025). Comparative analysis of maternal and neonatal outcomes in early versus late onset pre-eclampsia: a prospective study from northern India. Int J Reprod Contracept Obstet Gynecol.

[16] Susilo SA, Pratiwi KN, Fattah ANA, Irwinda R, Wibowo N (2014). Determinants of low APGAR score among preeclamptic deliveries in Cipto Mangunkusumo Hospital: a retrospective cohort study. Med J Indonesia.

[17] van Esch JJ, van Heijst AF, de Haan AF, van der Heijden OW (2017). Early-onset preeclampsia is associated with perinatal mortality and severe neonatal morbidity. J Matern Fetal Neonatal Med.

